# Dose–response tests and semi-field evaluation of lethal and sub-lethal effects of slow release pyriproxyfen granules (Sumilarv^®^0.5G) for the control of the malaria vectors *Anopheles gambiae* sensu lato

**DOI:** 10.1186/1475-2875-12-94

**Published:** 2013-03-14

**Authors:** Oscar Mbare, Steven W Lindsay, Ulrike Fillinger

**Affiliations:** 1 icipe-Thomas Odhiambo Campus, Mbita, Kenya; 2 Disease Control Department, London School of Hygiene & Tropical Medicine, London, UK; 3 School of Biological and Biomedical Sciences, Durham University, Durham, UK

**Keywords:** Pyriproxyfen, Sumilarv^®^0.5G, Malaria, Larval source management, *Anopheles gambiae* s.s., *Anopheles arabiensis*

## Abstract

**Background:**

Recently research has shown that larviciding can be an effective tool for integrated malaria vector control. Nevertheless, the uptake of this intervention has been hampered by the need to re-apply larvicides frequently. There is a need to explore persistent, environmentally friendly larvicides for malaria vector control to reduce intervention efforts and costs by reducing the frequency of application. In this study, the efficacy of a 0.5% pyriproxyfen granule (Surmilarv^®^0.5G, Sumitomo Chemicals) was assessed for the control of *Anopheles gambiae* sensu stricto and *Anopheles arabiensis*, the major malaria vectors in sub-Saharan Africa.

**Methods:**

Dose–response and standardized field tests were implemented following standard procedures of the World Health Organization’s Pesticide Evaluation Scheme to determine: (i) the susceptibility of vectors to this formulation; (ii) the residual activity and appropriate retreatment schedule for field application; and, (iii) sub-lethal impacts on the number and viability of eggs laid by adults after exposure to Sumilarv^®^0.5G during larval development.

**Results:**

*Anopheles gambiae* s.s. and *An. arabiensis* were highly susceptible to Sumilarv^®^0.5G. Estimated emergence inhibition (EI) values were very low and similar for both species. The minimum dosage that completely inhibited adult emergence was between 0.01-0.03 parts per million (ppm) active ingredient (ai). Compared to the untreated control, an application of 0.018 ppm ai prevented 85% (95% confidence interval (CI) 82%-88%) of adult emergence over six weeks under standardized field conditions. A fivefold increase in dosage of 0.09 ppm ai prevented 97% (95% CI 94%-98%) emergence. Significant sub-lethal effects were observed in the standardized field tests. Female *An. gambiae* s.s. that were exposed to 0.018 ppm ai as larvae laid 47% less eggs, and females exposed to 0.09 ppm ai laid 74% less eggs than females that were unexposed to the treatment. Furthermore, 77% of eggs laid by females exposed to 0.018 ppm ai failed to hatch, whilst 98% of eggs laid by females exposed to 0.09 ppm ai did not hatch.

**Conclusion:**

*Anopheles gambiae* s.s. and *An. arabiensis* are highly susceptible to Sumilarv^®^0.5G at very low dosages. The persistence of this granule formulation in treated habitats under standardized field conditions and its sub-lethal impact, reducing the number of viable eggs from adults emerging from treated ponds, enhances its potential as malaria vector control tool. These unique properties warrant further field testing to determine its suitability for inclusion in malaria vector control programmes.

## Background

Malaria control interventions with long-lasting insecticidal nets (LLINs) and indoor residual spraying (IRS) have resulted in substantial reductions of malaria cases in sub-Saharan Africa [1,2]. Since both LLINs and IRS target the fraction of the vector population that enter houses [3,4] their efficacy is threatened by vectors developing resistance to insecticides used indoors [5-7] and behavioural adaptations where vectors shift their biting patterns to bite in early evening and in the morning when people are out of the nets [8,9]. There has also been a shift in the vector species’ composition in parts of East Africa with LLINs dramatically reducing the numbers of largely endophilic *Anopheles gambiae* s.s. but having little or no impact on *Anopheles arabiensis* that tends to bite and rest outdoors [10-13] resulting in *An. arabiensis* becoming the dominant vector. Since IRS and LLINs cannot totally suppress malaria transmission there is a growing interest in the use of additional tools in an integrated vector management approach [14-18].

Larval source management has been re-evaluated for malaria control [19-24], with results indicating the added benefit larval control could have when used together with interventions that target adult mosquitoes [14,15,25]. One of the advantages of larval source management is that it targets the aquatic stages of the vectors thus controlling both indoor and outdoor biting and resting and insecticide resistant mosquitoes [26]. Commercially available chemical larvicides and microbials are highly effective in the control of the major malaria vectors of sub-Saharan Africa [20,24,27-33]. However, relatively few studies evaluated them under operational conditions [15,23,34-37] and a major limitation is their short activity under most environmental conditions, frequently requiring weekly re-application [20,21,34,38]. Larvicide and labour are the major costs in large-scale larval control programmes and these could be substantially reduced if re-application intervals could be reduced without jeopardizing the impact of the intervention [39]. In addition, the toxic effects of chemical-based larvicides to non-target aquatic insects limits their use for regular larviciding programmes [40,41].

Sumilarv^®^0.5G (Sumitomo Chemicals) is a granule insecticide developed for mosquito control. The active ingredient is pyriproxyfen (4-phenoxyphenyl (RS)-2-(2-pyridyloxy) propyl ether), a juvenile hormone analogue that acts as an insect growth regulator [42]. Pyriproxyfen generally inhibits adult emergence of target insects [43-45]. However it also has delayed effects on female reproduction of adult mosquitoes exposed to sub-lethal doses at the larval [46,47] or adult stage [48,49]. Sumilarv^®^0.5 has exceptional residual activity of up to six months for the control of *Aedes, Culex* and *Anopheles* mosquitoes in their natural breeding habitats [44,45,49,50]. Furthermore, pyriproxyfen has been evaluated as a safe insecticide for application in drinking water [51] with minimal impacts on non-target aquatic insects and the environment [52-56]. Nevertheless, Sumilarv^®^0.5G has never been evaluated for the control of immature stages of *An. gambiae* s.l., the major malaria vector in sub-Saharan Africa.

The objectives of the present study were to evaluate the efficacy of this granular formulation of pyriproxyfen for the control of *An. arabiensis* and *An. gambiae* s.s. by determining: (i) the minimum effective dose in dose–response tests; (ii) the optimum application dose to be applied under field conditions; (iii) the residual period of the optimum dose; and, (iv) the effects of sub-lethal doses on egg production and larval hatching. All tests were based on the World Health Organization Pesticide Evaluation Scheme (WHOPES) guidelines for laboratory and field testing of mosquito larvicides [57].

## Methods

### Study area

The study was conducted at the International Centre of Insect Physiology and Ecology-Thomas Odhiambo Campus (*icipe*-TOC) in Mbita (0° 26΄ 06.19” S; 34° 12΄ 53.13” E) close to Lake Victoria, Western Kenya (altitude 1,137 m). Here, the major malaria vectors are *An. arabiensis* with a small number of *An. gambiae* s.s. and *Anopheles funestus*[58]. The area is characterized by a tropical climate with an average annual minimum temperature of 16°C and an average maximum temperature of 28°C (*icipe*-TOC meteorological station data for 2010 to 2012). The area experiences two major rainy seasons, the long rains between March and June and the short rains between October and December. The average annual rainfall for 2010 to 2012 was 1,150 mm (*icipe*-TOC meteorological station).

### Mosquitoes

Both laboratory and standardized field tests used insectary-reared third instar larvae of *An. arabiensis* and *An. gambiae* s.s. (Mbita strains). Larvae were reared in round plastic tubs (diameter 60 cm) filled with water (5 l, 5 cm high) from Lake Victoria filtered through a charcoal-sand filter. Mosquito larvae were fed with fish food (Tetramin©Baby) twice daily. Third instar mosquito larvae were selected from different tubs so that the larvae were of a similar range in size in each tub tested [59]. Mosquito larvae were reared at ambient climate and light conditions in a netting-screened greenhouse with an average daily temperature of 27°C, an average 76% relative humidity and a natural 12 hours of dark and 12 hours of light cycle.

### Insecticide

Sumilarv^®^0.5G was provided by the manufacturer Sumitomo Chemicals Company, Japan, for all tests. It is a granular formulation containing 0.5% active ingredient (weight: weight).

### Dose–response tests

Tests were done in the shade, under ambient climate and light conditions in a netting-screened greenhouse. Prior to the dose–response tests, range-finding tests were implemented by exposing test larvae to a wide range of test concentrations and a control. This served to find the activity range of the insecticide for each test species. Concentrations between 10 parts per million (ppm) active ingredient (ai) and 0.0000001 ppm ai were tested. After determining the emergence inhibition (EI) of the larvae in the wider range, nine concentrations were chosen, yielding between 10% and 95% EI in the range-finding tests in order to determine the EI_50_, EI_90_ and EI_99_ in dose response bioassays. The following concentrations were tested: 0.005 ppm ai, 0.001 ppm ai, 0.0005 ppm ai, 0.0001 ppm ai, 0.00007 ppm ai, 0.00004 ppm ai and 0.00001 ppm ai, 0.000005 ppm ai, 0.000001 ppm ai.

A stock solution was prepared by grinding the granular formulation into a very fine powder following the procedure of Sihuincha and others [49]. Using a pestle and mortar, 5 g of Sumilarv^®^0.5G (25 mg ai) was ground and added to 500 ml of non-chlorinated tap water. This gave a stock solution of 10,000 ppm Sumilarv^®^0.5G (50 ppm ai). The mouth of the vial was covered with aluminium foil and the solution left to agitate for one hour on a shaker. Since Sumilarv^®^0.5G is a slow release formulation the mixture was left overnight to allow the active ingredient to be released into solution. In the morning the mixture was again agitated on a shaker for 30 minutes to prepare a homogenous mixture since some of the inert ingredients of the formulation (potentially still containing some active ingredient) had settled overnight. Serial dilutions were made immediately after shaking in non-chlorinated tap water to produce the test concentrations.

*Anopheles arabiensis* and *An. gambiae* s.s. were evaluated in parallel. Each test concentration and a control were replicated four times per round per mosquito species. Two hundred ml of each test solution was set up in 300 ml plastic cups. Three rounds of tests were implemented. Separate batches of 25 insectary-reared third instar larvae of both test species were introduced into each test concentration and the control (non-chlorinated tap water). Thus in total 300 larvae of each species were tested per test concentration and control (total of 3000 larvae). Larvae were fed with Tetramin© Baby fish food every 24 hours and cups covered with netting to prevent any emerging adults from escaping. The number of live and dead larvae, pupae and adults was recorded every 24 hours for 10 days. Live pupae from each cup were transferred into a separate cup with approximately 20 ml of water from the respective cup of collection. These cups were covered with netting and pupae monitored for emergence. Separate pipettes were used to collect pupae from treated and control cups to avoid cross-contamination.

### Standardized field tests

**Figure 1:**
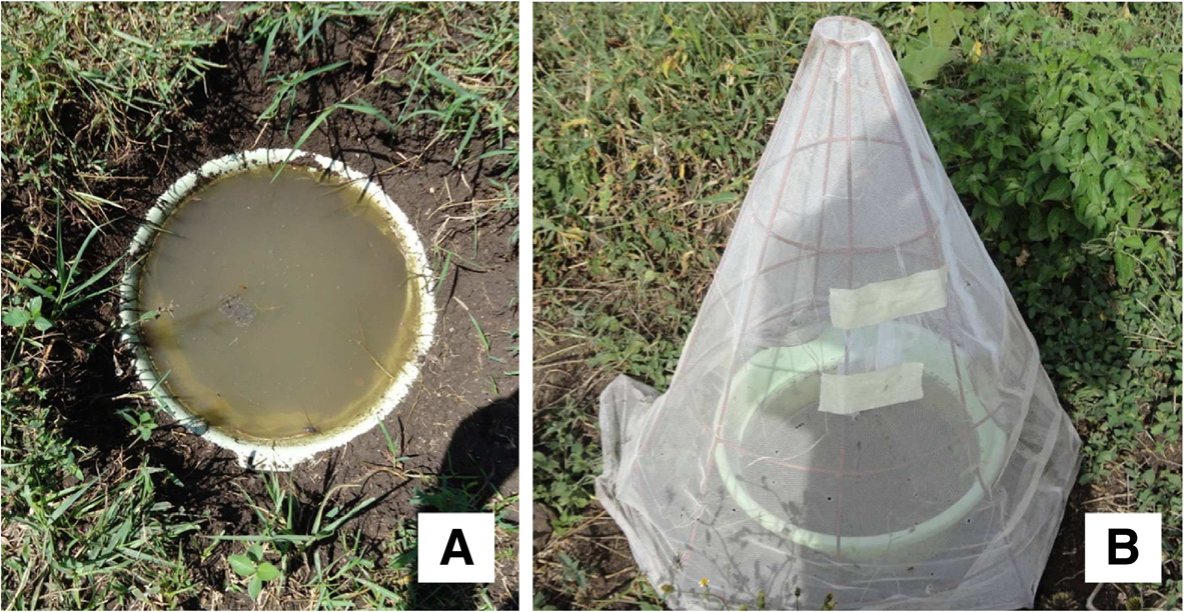
**Set-up of standardized field test. **(**A**) Enamel-coated bowl sunk into the ground and filled with water and soil to simulate a natural pond. (**B**) Netting-covered emergence trap on top of a pond to prevent escape of emerged adults.

Standardized field tests [57] were carried out in an open field with grass approximately 3 cm in height between October 2011 and March 2012. Thirty artificial ponds were set up in an open field by sinking enamel-coated bowls (diameter 42 cm, depth 10 cm) into the ground (Figure 1A). Ponds were arranged 2 m apart in six rows. Each bowl was filled with 8 l of non-chlorinated tap water. Into each pond 2 l of soil collected from the surrounding field was added and mixed well to resemble a natural habitat. Batches of 50 insectary-reared third instar larvae were introduced into each pond. Sumilarv^®^0.5G treatment was applied after introduction of larvae. Treatment of the ponds was allocated randomly using a lottery system. In each treatment round, 10 of the ponds served as untreated controls; in five of them *An. arabiensis* were introduced and in the other five *An. gambiae* s.s. Two application rates of Sumilarv^®^0.5G were tested per mosquito species. The application rate was based on the surface area of the water, which was 0.14 m^2^ per pond. Sumilarv^®^0.5G was spread evenly over the entire water surface by hand. Five ponds were treated with 1 mg ai per m^2^ (equalling 0.018 ppm ai considering the volume of 8 l of water) while five other ponds were treated with 5 mg ai per m^2^ (or 0.09 ppm ai) per mosquito species. A netting-covered emergence trap was placed on top of each pond to prevent wild mosquitoes from laying eggs in the sites and to prevent the escape of any emerging adult mosquitoes (Figure 1B). The residual activity of Sumilarv^®^0.5G was evaluated by introducing new batches of 50 insectary-reared third instar larvae into each pond at weekly intervals. After one week all the larvae had either emerged as adults or died. The efficacy of Sumilarv^®^0.5G was evaluated for six weeks. This experiment was implemented three times (referred to as rounds in the analyses).

To assess larval mortality, the number of larvae present in each habitat was counted daily. First, the emergence trap over each pond was assessed for the presence of any newly emerged adults and any adults collected with an aspirator and placed into a disposable cup covered with netting. Any pupae in the ponds were transferred into plastic cups holding 50 ml of the water from the respective pond. Pupae collections were done in the morning and evening so that any emergence or emergence inhibition could be recorded daily in the laboratory.

To monitor environmental parameters that may influence the efficacy of the insecticide, daily data on turbidity and pH of water in each pond was collected. Ponds were visually categorized into clear (ground visible) or turbid ponds. The water pH was measured using a pH meter (Phywe International, Germany).

### Sub-lethal effects

Tests to assess the impact of sub-lethal doses of Sumilarv^®^0.5G were carried out under ambient conditions in a netting-screened greenhouse. The number of eggs laid and the number of eggs hatched (number of offspring produced) per adult mosquito that emerged from treated ponds were compared to that of the adults that emerged from the untreated ponds in standardized field tests. All pupae used in these tests were collected from the ponds in week six of each test round. Emerged adults were maintained with 6% glucose solution *ad libitum*. When the adults were two to four days old they were blood-fed twice on a human arm on two successive days. A single gravid mosquito was introduced into each cage with an oviposition cup (diameter = 7 cm) containing 100 ml of non-chlorinated tap water. The number of eggs laid by each mosquito overnight and the number of eggs hatched over one week were counted. Sub-lethal effects of the treatment dosage of 1 mg ai per m^2^ were tested with 20 individual females per round of semi-field test for *An. arabiensis* and *An. gambiae* s.s., respectively (total 3 × 20 = 60 females per species). Due to the persistent high immature mortality of the 5 mg ai per m^2^ treatment only 10 females per species and round could be tested (total 3 × 10 = 30 females per species).

### Statistical analyses

Data analyses were done with SPSS statistical software version 19. All data from the replicates of the dose–response tests were pooled by doses for each mosquito species for the estimation of the EI_50_, EI_90_ and EI_99_ values using the log dosage-probit regression analysis with the test dosages as covariates and species as factors in the model. Relative median potency estimates were used to compare the susceptibility of the two species. Generalized estimating equations (GEE) were used to estimate the overall emergence inhibition of the two Sumilarv^®^0.5G dosages for the six weeks treatment period in standardized field tests. The number of successful emerged adults was the dependent variable and was fitted to a negative binomial distribution with a log-link function and an exchangeable correlation matrix. The treatments, test rounds, mosquito species, water turbidity (clear, turbid), water pH (grouped in two categories: pH < 8, pH ≥8) and the occurrence of rain during the test week (no rain, rain) were added to the model as fixed factors. Since the same pond was evaluated repeatedly for larval mortality over the six-week period, the unique pond ID was included as the repeated measures variable. Interaction terms were included in the model between treatments and turbidity, treatments and pH, and treatments and rain. GEE models were also used to estimate the impact of sub-lethal concentrations on the number of eggs laid and the number of eggs that hatched from emerged *An. gambiae* s.s. adults. The parameter estimates of the GEE models were used to calculate the weekly mean adult emergence, mean number of eggs laid per female and mean number of laid eggs that hatched into larvae and the associated 95% confidence intervals (CIs) by removing the intercept from the models. For the calculation of percent reduction the weekly emergence inhibition in the treated ponds was corrected using Abbott’s formula based on emergence in the untreated ponds as denominator [60]. Percent reduction was therefore calculated as follows:

$$\%\;\text{treatment}\;\mathrm{EI}=\frac{\left[\%\;\text{untreated}\;\mathrm{EI}-\%\;\text{treated}\;\mathrm{EI}\right]\times 100\%}{\%\;\text{untreated}\;\mathrm{EI}}$$

## Results

### Dose–response tests

**Figure 2:**
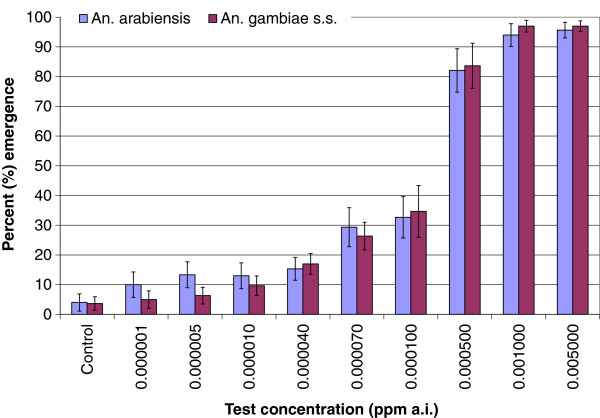
**Average percent emergence inhibition (error bars: 95% confidence intervals) of *Anopheles arabiensis *and *Anopheles gambiae *s.s. in response to increasing concentrations (ppm ai) of Sumilarv^®^0.5G.**

The dose–response tests showed that Sumilarv^®^0.5G affected adult mosquito emergence in *An. arabiensis* and *An. gambiae* s.s. at very low and over a very wide range of concentrations (0.000001-0.005 ppm ai). Data from the three rounds of dose–response tests showed similar trends in emergence inhibition for each species and were, therefore, pooled per dose (Figure 2) to estimate emergence inhibition (EI) rates; EI _50_, EI_90_ and EI_99_ (Table 1). The minimum dosage that completely inhibited adult emergence was estimated to be between 0.01-0.03 ppm ai (Table 1). *Anopheles arabiensis* and *An. gambiae* s.s. were equally susceptible to Sumilarv^®^0.5G.

**Table 1:** **Estimated doses (ppm ai) of Sumilarv^®^0.5G for 50%, 90% and 99% emergence inhibition (EI) in *Anopheles gambiae *s.s. and *Anopheles arabiensis***

	***Anopheles arabiensis***	***Anopheles gambiae *s.s.**
	**ppm ai**	**ppm ai**
**EI**_**50 **_**(95%CI)**	0.00012 (0.00009-0.00016)	0.00013 (0.00010-0.00017)
**EI**_**90 **_**(95%CI)**	0.00248 (0.00154-0.00450)	0.00139 (0.00092-0.00232)
**EI**_**99 **_**(95%CI)**	0.02860 (0.01379-0.07296)	0.00973 (0.00526-0.02159)

### Standardized field tests

**Figure 3:**
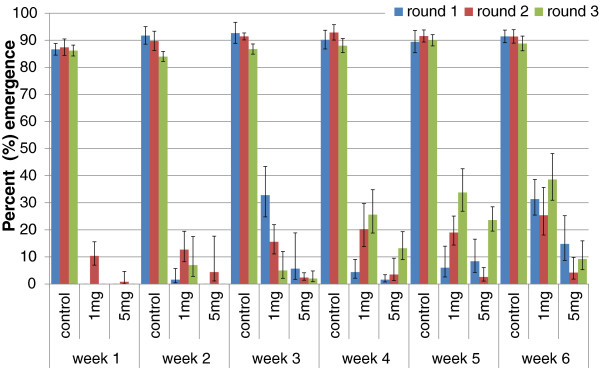
**Mean adult emergence (error bars: 95% confidence intervals) of *Anopheles gambiae *s.l. in standardized field tests after application of 1 mg or 5 mg ai per m**^**2 **^**Sumilarv^®^0.5G in artificial ponds.**

**Figure 4:**
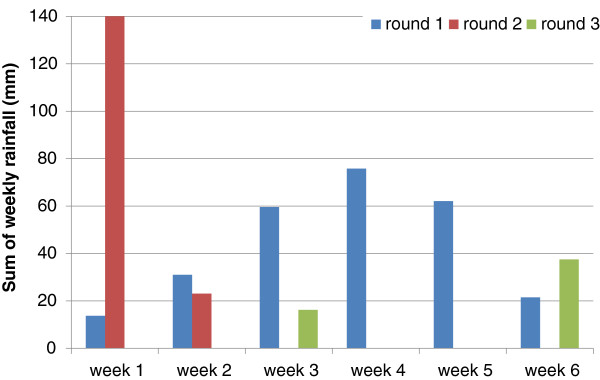
Weekly rainfall during the three rounds of standardized field tests.

There was no difference in adult emergence from treated ponds between *An. arabiensis* and *An. gambiae* s.s. (p=0.3) and data for both species were pooled for analysis. The weekly adult emergence per round from the treated and untreated ponds is shown in Figure 3 and emergence inhibition calculated in Table 2. Complete emergence inhibition was observed for two weeks in rounds one and three of the high treatment dose of 5 mg ai per m^2^ (0.09 ppm ai). However at the lower dosage of 1 mg ai per m^2^ (0.018 ppm ai) which corresponded with the minimum effective dosage established in the dose–response tests complete emergence inhibition was only observed in week one in round one and three. Ponds treated at 5 mg ai per m^2^ provided better residual impact than the lower treatment dosage of 1 mg ai per m^2^ (Figure 3 and Table 2). Adjusting for other factors the GEE model estimated that Sumilarv^®^0.5G inhibited 85% of adult emergence over a period of six weeks at an application dose of 1 mg ai per m^2^ and 97% at a dose of 5 mg ai per m^2^ compared to emergence from untreated ponds (Table 3). The overall impact of 5 mg ai per m^2^ on inhibiting emergence was significantly higher than the impact of 1 mg ai per m^2^ (p<0.001). Despite consistent rainfall during the first round of the standardized field tests and occasional rainfall during the following two rounds (Figure 4), rain did neither affect the emergence of adults from control and treatment ponds nor the impact of the treatments (Table 3). There were also no main effects of water turbidity or pH on adult emergence but interactions were identified between the treatments and water turbidity, and the treatments and water pH. Turbid water and high pH reduced the impact of the treatments leading to slightly higher adult emergence from treatment ponds under these conditions (Table 3). The impact of the interactions can be calculated by multiplication of the odds ratios [61]. This means for example emergence inhibition was 85% at 1 mg ai per m^2^ when ponds were clear and had a pH <8, emergence inhibition was reduced to 79% when the same treatment pond was turbid with a pH <8 and to 74% when the same treatment pond was turbid and had a pH ≥8. Similarly for the 5 mg ai per m^2^ ponds in round one, overall emergence inhibition is 97% when treatment ponds are clear with pH <8, emergence inhibition is reduced to 95% when the treatment ponds are turbid with pH <8 and further reduced to 90% when the treatment ponds are turbid and with pH ≥8.

**Table 2:** **Weekly percent emergence inhibition (95% CI) of *Anopheles gambiae *s.l. from treated ponds**

		**Week 1**	**Week 2**	**Week 3**	**Week 4**	**Week 5**	**Week 6**
1 mg ai per m^2^							
	Round 1	100	98 (94–99)	65 (55–72)	95 (90–98)	93 (85–97)	66 (59–71)
	Round 2	88 (83–92)	86 (76–90)	83 (76–88)	78 (69–85)	79 (73–84)	72 (62–80)
	Round 3	100	92 (80–97)	94 (86–98)	71 (62–78)	62 (54–69)	57 (47–64)
5 mg ai per m^2^							
	Round 1	100	100	94 (80–98)	98 (96–99)	91 (82–95)	84 (73–90)
	Round 2	99 (95–100)	95 (81–99)	97 (96–98)	96 (90–99)	97 (94–99)	95 (90–98)
	Round 3	100	100	98 (95–99)	85 (79–89)	74 (69–78)	90 (83–94)

**Table 3:** Multivariable analyses (GEE) of factors affecting the emergence of adult malaria vectors over a six week period from artificial ponds treated with Sumilarv^®^0.5G

**Explanatory variable**	**OR**	**95% CI**	**p**
**Treatment**			
1 mg ai per m^2^	0.03	0.02-0.05	<0.0001
5 mg ai per m^2^	0.15	0.12-0.18	<0.0001
control	1		
**Round**			
round 3	1.19	1.00-1.41	0.050
round 2	1.03	0.78-1.34	0.859
round 1	1		
**Vector species**			
*An. arabiensis*	0.95	0.86-1.05	0.278
*An. gambiae* s.s.	1		
**Water turbidity**			
turbid	1.01	0.95-1.07	0.765
clear	1		
**Water pH**			
≥ 8	0.99	0.91-1.08	0.820
< 8	1		
**Rain during test week**			
rain	1.05	0.92-1.20	0.449
no rain	1		
**Interaction between treatment and turbidity**			
5 mg ai per m^2^*turbid	1.93	1.12-3.26	0.017
5 mg ai per m^2^*clear	1		
1 mg ai per m^2^*turbid	1.40	1.08-1.79	0.011
1 mg ai per m^2^*clear	1		
**Interaction between treatment and pH**			
5 mg ai per m^2^*pH≥8	1.90	1.13-2.85	0.002
5 mg ai per m^2^*pH<8	1		
1 mg ai per m^2^*pH≥8	1.25	1.06-1.47	0.008
1 mg ai per m^2^*pH<8	1		
**Interaction between treatment and rain**			
5 mg ai per m^2^*rain	1.23	0.89-1.69	0.211
5 mg ai per m^2^*no rain	1		
1 mg ai per m^2^*rain	0.87	0.70-1.07	0.870
1 mg ai per m^2^*no rain	1		

### Sub-lethal effects

The impact of sub-lethal effects could not be evaluated for *An. arabiensis* that emerged from pupae since neither females from untreated ponds nor females from treated ponds laid eggs, possibly due to unsuitable mating conditions provided for this species [62]. Exposure of *An. gambiae* s.s. to both Sumilarv^®^0.5G dosages during the larval stage resulted in: (i) a reduced probability of the adult female laying eggs; (ii) reduced mean number of eggs laid per female; and, (iii) reduced mean number of eggs that hatched into larvae (Table 4). Treatment rounds were not significantly different (p=0.687), and data for all rounds for *An. gambiae* s.s. were pooled for analysis. Mosquitoes that emerged from treated ponds were 65-68% less likely to lay eggs compared to mosquitoes that emerged from untreated ponds. The mean number of eggs laid per female *An. gambiae* s.s. was reduced by 47% from females emerging from ponds treated at 1 mg ai per m^2^ and by 74% from females emerged from ponds treated at 5 mg ai per m^2^ compared to that in the untreated controls (Table 4). The impact of the higher dosage was twice the impact measured from the lower dosage (odds ratio (OR) 2.1, 95% CI 1.2-3.7, p=0.02). Furthermore, it was 90% less likely for an egg to hatch that was laid by a female exposed to the higher Sumilarv^®^0.5G dosage compared to eggs laid by females that emerged from low dosage ponds (OR=0.10, 95% CI 0.04-0.23, p<0.0001). The probability of an egg hatching was reduced by 77% for eggs laid by a female exposed to the lower treatment dosage and 98% for eggs laid by a female exposed to the higher dosage as compared to eggs in females that emerged from the untreated control ponds.

**Table 4:** **Sub-lethal effects of Sumilarv^® ^0.5G on egg laying and hatching of *Anopheles gambiae *s.s.**

	**Control**	**1 mg ai per m**^**2**^	**5 mg ai per m**^**2**^
Number of females exposed	60	60	30
Number of females that laid eggs	43	27	14
Mean number of eggs/female (95% CI)	43.8 (35.6-53.8)	23.1 (16.5-32.3)	11.2 (6.9-18.2)
Mean number eggs/female hatched (95% CI)	37.4 (30.5-45.8)	8.7 (6.0-12.4)	0.8 (0.4-1.8)

## Discussion

*Anopheles arabiensis* and *An. gambiae* s.s. were equally and highly susceptible to Sumilarv^®^0.5G under laboratory and standardized field conditions. Sumilarv^®^0.5G inhibited over 80% of the total adult emergence over a period of six weeks at both application dosages. However, weekly emergence rates increased steadily over the six-week test period at the lower dosage that corresponded with the EI_99_ in the laboratory and weekly emergence inhibition was frequently lower than the 80% that is recommended by WHOPES for successful immature control [57]. Laboratory tests were conducted under standardized conditions without major abiotic and biotic influences and therefore EI values represent only minimum dosages. Application rates frequently have to be increased up to several times the minimum dose to obtain sufficient immature control under field conditions [57,63]. The higher dosage of 5 mg ai per m^2^ or 0.09 ppm ai inhibited well over 80% of adult emergence in all but one test week. This dosage was 4.5 times the average EI_99_ in the laboratory. Further field tests to establish the optimum dose for operational control in a variety of different habitats are necessary but based on the results presented here it is likely that the optimum dosage lies between the two tested here and therefore coincides with the maximum dosage recommended by the manufacturer (0.05 ppm ai) for operational control of other mosquito species [64].

The estimated emergence inhibition rates from the dose–response tests were four times higher than those previously reported by Kawada and his colleagues [65] for *An. gambiae,* but within the range of rates estimated for *Culex* and *Aedes* species [66-70]. These differences may arise from the different pyriproxyfen formulations used in separate studies [71], but also from the material of the test containers [44]. Kawada and colleagues used a 5% emulsifiable concentrate formulation while in the present study a granular formulation was used and had to be crushed in a mortar for the laboratory tests, which might have not led to an equal amount of active ingredients being released into the stock solution. Also, in the present study plastic cups were used for bioassays while Kawada and his colleagues used aluminium cups. There is a concern that the active ingredient pyriproxyfen adheres to plastic [72] leading to a longer residual effect from such treated containers due to a continuous slow release from the plastic [54]. In the short term however, plastic might reduce the amount of active ingredient in the water, which could be responsible for the higher estimates of EI concentrations found in this study. The extremely low concentrations of active ingredient needed for the control of mosquitoes with Sumilarv^®^0.5G is worth noting. The estimated effective dose of pyriproxyfen is approximately 10 times lower than those reported for microbial larvicides [20,21]. This is not surprising since pyriproxyfen is a juvenile hormone analogue, and insect hormones, like all hormones, operate at extremely low concentrations as chemical messengers [70,73]. Thus, far smaller quantities of Sumilarv^®^0.5G would be required for larviciding programmes compared to microbial larvicides, thereby helping to lower costs associated with transporting and storing larvicides [39].

The residual impact of Sumilarv^®^0.5G on *An. gambiae* s.l. emergence observed here corresponds well with reports from previous studies on other mosquito species [44,67,74] but application dosages required to achieve the same effect seem slightly higher for *An. gambiae* s.l. Sumilarv^®^0.5G at 0.02 ppm ai and 0.05 ppm ai provided almost complete emergence inhibition of *Aedes aegypti*, *Aedes albopictus* and *Aedes taeniorhynchus*, *Culex nigripalpus* and *Anopheles quadrimaculatus* for six weeks under standardized field conditions [74]. This slow-release formulation has even been shown to exhibit prolonged residual activity for control of *Aedes* larvae even when the treatments were diluted by using replacement of treated water with untreated water in the treated containers [44,75]. Similarly, here it was observed that rainfall did not negatively affect the impact of the treatments. Exceptional performance of Sumilarv^®^0.5G was reported for the control of *Anopheles culicifacies* in confined gem pits in Sri Lanka [45] where a single application of pyriproxyfen at 0.01 ppm ai was sufficient to inhibit adult emergence for approximately six months. Similarly, Sihuincha and colleagues [49] reported complete emergence inhibition of *Ae. aegypti* for five months from water tanks in Peru at an application rate of Sumilarv^®^0.5G of 0.05 ppm ai. Overall it can be concluded from previous work that the efficacy and residual activity of different pyriproxyfen-containing products depends on the formulation, dose, habitat types treated, prevailing weather conditions and target mosquito species [53,67,74].

The current study showed that the efficacy of Sumilarv^®^0.5G is reduced in turbid water and water with a pH ≥8. Water is turbid because it carries a suspension of fine particles of both organic and inorganic matter in the water column. Some of the turbidity observed here might have been due to algae and bacteria growth in the established habitats, which in turn might have increased the water pH. It is possible that the active ingredient, pyriproxyfen, is adsorbed onto particles in the water column and was less accessible to larvae. Turbidity and pH of aquatic habitats are important parameters that are associated with the abundance, development and survival of *Anopheles* larvae [76]. *Anophele*s larvae are known to exploit aquatic habitats with varying degrees of water turbidity and pH [76,77]. Suspended particles including algae in the water column in turbid ponds provide mosquitoes with food that enhances their development and survival thus increase emergence from turbid ponds [78,79]. Mulligan and Schaefer [80] found pyriproxyfen to adsorb onto organic matter which might have been responsible for larvae to be exposed to reduced doses. This needs to be considered and monitored in field operations where it might be necessary to increase the application dose or reduce retreatment intervals to ensure a consistent emergence inhibition above 80% as recommended by WHOPES [57].

An added benefit to the direct effect of Sumilarv^®^0.5G on immature stages were the sub-lethal effects that affected the offspring of adult females that successfully emerged from treated ponds. At 5 mg ai per m^2^ the reproduction of females was reduced by well over 90%. Similar effects of insect growth regulators have been shown for *Aedes* and *Culex*[46,47,81]. The laying of non-viable eggs by female *An. gambiae s.s.* emerging from treated ponds might further extend the efficacy and residual effect of pyriproxyfen, and may help further reduce intervention costs by extending the retreatment intervals. It would be particularly helpful in the context of an auto-dissemination strategy [82] of Sumilarv^®^0.5G where potentially only sub-lethal doses are transferred to a habitat by female gravid mosquitoes. The delayed sub-lethal effects of insect growth regulators were also shown to affect the sex ratio and to reduce blood-feeding rates in exposed mosquitoes [47,83]. Similar effects were shown for adults exposed to pyriproxifen [48,49,84]. Ohashi and colleagues [84] demonstrated that *An. gambiae* s.s. was completely sterilized, with no female laying eggs after exposure to pyriproxyfen-treated nets. Insect growth regulators have been shown to suppress ovarian development and egg development in mosquitoes [85,86]. Judson and de Lumen [85] showed that exposure of *Ae. aegypti* females to juvenile hormone analogues suppressed egg development by inhibiting development of ovarian follicles. Fournet and colleagues [86] similarly showed that the ovarian development of *Ae. aegypti* females that emerged from larvae exposed to insect growth regulators was affected.

As with every insecticide it is important to be cautious about using pyriproxyfen formulations as a stand-alone intervention since tolerance to pyriproxyfen has been found in Diptera [87,88]. It is also of concern to know whether the progeny of gravid females that are exposed to sub-lethal level doses of pyriproxyfen and survive have greater tolerance to pyriproxyfen than other mosquitoes. If this is the case, resistance may spread.

Pyriproxyfen exhibits favourable characteristics for utilization as a larvicide for mosquito control. The recommended application rate in drinking water limit of 300 ppb (0.3 ppm) [51] is several folds higher than the recommended dose of 0.01-0.05 ppm [64] for mosquito control and also has minimal environmental impacts at recommended rates for mosquitoes [52,53].

## Conclusion

*Anopheles arabiensis* and *An. gambiae* s.s. are highly susceptible to Sumilarv^®^0.5G at very low dosages. The persistence of Sumilarv^®^0.5G in treated habitats under standardized field conditions and its sub-lethal impact, reducing the number of viable eggs from adults emerging from treated ponds, enhances its potential as a malaria vector control tool in integrated vector management strategies. These unique properties of Sumilarv^®^0.5G warrant further field testing in a range of natural *An. gambiae* s.l. larval habitats and under operational conditions to recommend if and how this insect growth regulator could be included in vector control programmes for malaria control in sub-Saharan Africa.

Based on the results of this study the maximum dosage recommended by the manufacturer for other mosquito species of 0.05 ppm ai is recommended as the minimum dosage for further field testing for *An. gambiae* s.l control. Although the residual effect observed for the test concentrations lasted for a six-week period, initially a shorter retreatment interval should be evaluated under natural conditions where habitat types and water quality are highly heterogeneous and might affect the residual activity. Furthermore, the estimation of retreatment intervals should also consider the probability of new habitats emerging during treatment cycles that could then harbour mosquito larvae that might successfully emerge before the target area receives another round of Sumilarv^®^0.5G application. Initial application cycles should be determined for the predominant habitat type in the target area, the season of application and the development time of immature vectors. In areas where temporary habitats dominate or areas with high rainfall an initial application cycle of two to three weeks should be tested whilst in areas of more semi-permanent to permanent habitats or during dry seasons a three to four-weekly application cycle might be appropriate for an initial field operation informed by a monitoring and evaluation programme.

## Competing interests

Sumitomo Chemicals, Japan, the commercial manufacturer of Sumilarv^®^0.5G, provided the insecticide for this study free of charge. Nevertheless, neither the manufacturer nor any of the funders of this work had any role in the design, analysis or interpretation of the results, nor in the drafting of the manuscript.

## Authors’ contributions

UF and SWL conceived the idea for this research. OM, SWL and UF developed the experimental design and protocols. OM implemented the experiments. OM and UF analysed the data and drafted the manuscript. All authors contributed to the final draft, read and approved the manuscript.

## References

[B1] Steketee RW Campbell CC Impact of national malaria control scale-up programmes in Africa: magnitude and attribution of effects Malar J 2010 9 299 10.1186/1475-2875-9-299 20979634 PMC2988827

[B2] Okumu FO Moore SJ Combining indoor residual spraying and insecticide-treated nets for malaria control in Africa: a review of possible outcomes and an outline of suggestions for the future Malar J 2011 10 208 10.1186/1475-2875-10-208 21798053 PMC3155911

[B3] Robert V Carnevale P Influence of deltamethrin treatment of bed nets on malaria transmission in the Kou valley, Burkina Faso Bull World Health Organ 1991 69 735 740 1786622 PMC2393314

[B4] Pinder M Jawara M Jarju LBS Kandeh B Jeffries D Lluberas MF Mueller J Parker D Bojang K Conway DJ Lindsay SW To assess whether indoor residual spraying can provide additional protection against clinical malaria over current best practice of long-lasting insecticidal mosquito nets in The Gambia: study protocol for a two-armed cluster-randomized trial Trials 2011 12 147 10.1186/1745-6215-12-147 21663656 PMC3121610

[B5] WHO Expert Committee on malaria, 892 2000 Geneva: WHO Technical Report Series 1 71 10892307

[B6] Kawada H Futami K Komagata O Kasai S Tomita T Sonye G Mwatele C Njenga SM Mwandawiro C Minakawa N Takagi M Distribution of a knockdown resistance mutation (L1014S) in Anopheles gambiae s.s. and Anopheles arabiensis in Western and Southern Kenya PLoS One 2011 6 e24323 10.1371/journal.pone.0024323 21931682 PMC3170322

[B7] Chouaibou M Etang J Brevault T Nwane P Hinzoumbe CK Mimpfoundi R Simard F Dynamics of insecticide resistance in the malaria vector Anopheles gambiae s.l. from an area of extensive cotton cultivation in Northern Cameroon Trop Med Int Health 2008 13 476 486 10.1111/j.1365-3156.2008.02025.x 18248566

[B8] Reddy MR Overgaard HJ Abaga S Reddy VP Caccone A Kiszewski AE Slotman MA Outdoor host seeking behaviour of Anopheles gambiae mosquitoes following initiation of malaria vector control on Bioko Island, Equatorial Guinea Malar J 2011 10 184 10.1186/1475-2875-10-184 21736750 PMC3146901

[B9] Faye O Konate L Mouchet J Fontenille D Ngayo SY Hebrard G Herve JP Indoor resting by outdoor biting females of Anopheles gambiae complex (Diptera: Culicidae) in the Sahel of Northern Senegal J Med Entomol 1997 34 285 289 9151491 10.1093/jmedent/34.3.285

[B10] Bayoh MN Mathias DK Odiere MR Mutuku FM Kamau L Gimnig JE Vulule JM Hawley WA Hamel MJ Walker ED Anopheles gambiae: historical population decline associated with regional distribution of insecticide-treated bed nets in western Nyanza Province Kenya. Malar J 2010 9 62 10.1186/1475-2875-9-62 PMC2838909 20187956

[B11] Russell TL Lwetoijera DW Maliti D Chipwaza B Kihonda J Charlwood JD Smith TA Lengeler C Mwanyangala MA Nathan R Knols BGJ Takken W Killeen GF Impact of promoting longer-lasting insecticide treatment of bed nets upon malaria transmission in a rural Tanzanian setting with pre-existing high coverage of untreated nets Malar J 2010 9 187 10.1186/1475-2875-9-187 20579399 PMC2902500

[B12] Sinka ME Bangs MJ Manguin S Coetzee M Mbogo CM Hemingway J Patil AP Temperley WH Gething PW Kabaria CW Okara RM Boeckel TV Godfray HCJ Harbach RE Hay SI The dominant Anopheles vectors of human malaria in Africa, Europe and the Middle East: occurrence data, distribution maps and bionomic precis Parasit Vectors 2010 3 117 10.1186/1756-3305-3-117 21129198 PMC3016360

[B13] Kitau J Oxborough RM Tungu PK Matowo J Magesa SM Bruce J Mosha FW Rowland MW Species shifts in the Anopheles gambiae complex: do LLINs successfully control Anopheles arabiensis? PLoS One 2012 7 e31481 10.1371/journal.pone.0031481 22438864 PMC3306310

[B14] Chanda E Masaninga F Coleman M Sikaala C Katebe C Macdonald M Baboo KS Govere J Manga L Integrated vector management: the Zambian experience Malar J 2008 7 164 10.1186/1475-2875-7-164 18752658 PMC2551620

[B15] Fillinger U Ndenga B Githeko A Lindsay SW Integrated malaria vector control with microbial larvicides and insecticide-treated nets in western Kenya: a controlled trial Bull World Health Organ 2009 87 655 665 10.2471/BLT.08.055632 19784445 PMC2739910

[B16] Beier JC Keating J Githure JI Macdonald MB Impoinvil DE Novak RJ Integrated vector management for malaria control Malar J 2008 7 Suppl 1 S4 10.1186/1475-2875-7-S1-S4 19091038 PMC2604879

[B17] WHO Global strategic framework for integrated vector management 2004 whqlibdoc.who.int/hq/2004/WHO_CDS_CPE_PVC_2004_2010.pdf

[B18] Clive S Integrated approach to malaria control Clin Microbiol Rev 2002 15 278 293 10.1128/CMR.15.2.278-293.2002 11932233 PMC118067

[B19] Bukhari T Knols BG Efficacy of Aquatain, a monomolecular surface film, against the malaria vectors Anopheles stephensi and An. gambiae s.s. in the laboratory Am J Trop Med Hyg 2009 80 758 763 19407120

[B20] Fillinger U Knols BG Becker N Efficacy and efficiency of new Bacillus thuringiensis var israelensis and Bacillus sphaericus formulations against Afrotropical anophelines in Western Kenya Trop Med Int Health 2003 8 37 47 10.1046/j.1365-3156.2003.00979.x 12535249

[B21] Majambere S Lindsay SW Green C Kandeh B Fillinger U Microbial larvicides for malaria control in the Gambia Malar J 2007 6 76 10.1186/1475-2875-6-76 17555570 PMC1899511

[B22] Geissbuhler Y Kannady K Chaki PP Emidi B Govella NJ Mayagaya V Kiama M Mtasiwa D Mshinda H Lindsay SW Tanner M Fillinger U Castro MC Killeen GF Microbial larvicide application by a large-scale, community-based program reduces malaria infection prevalence in urban Dar es Salaam Tanzania. PLoS ONE 2009 4 e5107 10.1371/journal.pone.0005107 PMC2661378 19333402

[B23] Fillinger U Lindsay SW Suppression of exposure to malaria vectors by an order of magnitude using microbial larvicides in rural Kenya Trop Med Int Health 2006 11 1629 1642 10.1111/j.1365-3156.2006.01733.x 17054742

[B24] Shililu JI Tewolde GM Brantly E Githure JI Mbogo CM Beier JC Fusco R Novak RJ Efficacy of Bacillus thuringiensis israelensis, Bacillus sphaericus and temephos for managing Anopheles in Eritrea J Am Mosq Control Assoc 2003 19 251 258 14524547

[B25] Shaukat AM Breman JG McKenzie FE Using the entomological inoculation rate to assess the impact of vector control on malaria parasite transmission and elimination Malar J 2010 9 122 10.1186/1475-2875-9-122 20459850 PMC2890672

[B26] Fillinger U Lindsay SW Larval source management for malaria control in Africa: myths and reality Malar J 2011 10 353 10.1186/1475-2875-10-353 22166144 PMC3273449

[B27] Seyoum A Abate D Larvicidal efficacy of Bacillus thuringiensis var. israelensis and Bacillus sphaericus on Anopheles arabiensis in Ethiopia World J Microb Biot 1997 13 21 24 10.1007/BF02770802

[B28] Karch S Manzambi ZA Salaun JJ Field trials with Vectolex (Bacillus sphaericus) and Vectobac (Bacillus thuringiensis (H-14)) against Anopheles gambiae and Culex quinquefasciatus breeding in Zaire J Am Mosq Control Assoc 1991 7 176 179 1895075

[B29] Karch S Asidi N Manzambi ZM Salaun JJ Efficacy of Bacillus sphaericus against the malaria vectors Anopheles gambiae and other mosquitoes in swamps and rice fields in Zaire J Am Mosq Control Assoc 1992 8 376 380 1361940

[B30] Majori G Ali A Sabatinelli G Laboratory and field efficacy of Bacillus thuringiensis var israelensis and Bacillus sphaericus against Anopheles gambiae s.l. and Culex quinquefasciatus in Ouagadougou, Burkina Faso J Am Mosq Control Assoc 1987 3 20 25 3504891

[B31] Skovmand O Bauduin S Efficacy of a granular formulation of Bacillus sphaericus against Culex quinquefasciatus and Anopheles gambiae in West African countries J Vector Ecol 1996 22 43 51 9221738

[B32] Ravoahangimalala O Thiery I Sinegre G Rice field efficacy of deltamethrin and Bacillus thuringiensis israelensis formulations on Anopheles gambiae s.s. in the Anjiro Region of Madagascar Bull Soc Vector Ecol 1994 19 169 174

[B33] Ragoonanansingh RN Njunwa KJ Curtis CF Becker N A field study of Bacillus sphaericus for the control of culicine and anopheline mosquito larvae in Tanzania Bull Soc Vector Ecol 1992 17 45 50

[B34] Fillinger U Kannady K William G Vanek MJ Dongus S Nyika D Geissbuhler Y Chaki PP Govella NJ Mathenge EM Singer BH Mshinda H Lindsay SW Tanner M Mtasiwa D de Castro MC Killeen GF A tool box for operational mosquito larval control: preliminary results and early lessons from the urban malaria control programme in Dar es Salaam, Tanzania Malar J 2008 7 20 10.1186/1475-2875-7-20 18218148 PMC2259364

[B35] Majambere S Pinder M Fillinger U Ameh D Conway DJ Green C Jeffries D Jawara M Milligan PJ Hutchinson R Lindsay SW Is mosquito larval source management appropriate for reducing malaria in areas of extensive flooding in the Gambia? A cross-over intervention trial Am J Trop Med Hyg 2010 82 176 184 10.4269/ajtmh.2010.09-0373 20133989 PMC2813154

[B36] Shililu J Mbogo C Ghebremeskel T Githure J Novak R Mosquito larval habitats in a semiarid ecosystem in Eritrea: impact of larval habitat management on Anopheles arabiensis population Am J Trop Med Hyg 2007 76 103 110 17255237

[B37] Barbazan P Baldet T Darriet F Escaffre H Djoda DH Hougard JM Impact of treatments with Bacillus sphaericus on Anopheles populations and the transmission of malaria in Maroua, a large city in a savannah region of Cameroon J Am Mosq Control Assoc 1998 14 33 39 9599321

[B38] Skovmand O Sanogo E Experimental formulations of Bacillus sphaericus and B. thuringiensis israelensis against Culex quinquefasciatus and Anopheles gambiae (Diptera: Culicidae) in Burkina Faso J Med Entomol 1999 36 62 67 10071494 10.1093/jmedent/36.1.62

[B39] Worrall E Fillinger U Large-scale use of mosquito larval source management for malaria control in Africa: a cost analysis Malar J 2011 10 338 10.1186/1475-2875-10-338 22067606 PMC3233614

[B40] Fortin C Maire A Leclair R The residual effect of temephos (Abate 4-E) on nontarget communities J Am Mosq Control Assoc 1987 3 282 288 2462615

[B41] Fales JH Spangler PJ Bodenstein OF Mills GD Jr Durbin CG Laboratory and field evaluation of Abate against a backswimmer (Notonecta undulata Say) (Hemiptera: Notonectidae) Mosq News 1968 28 77 81

[B42] Sumilarv product information http://www.olyset.net/vectorcontrol/sumilarv/

[B43] Kamimura K Arakawa A Field evaluation of an insect growth regulator, pyriproxyfen, against Culex pipiens Pallens and Culex tritaeniorhynchus Jap J Sanit Zool 1991 42 249 252

[B44] Vythilingam I Luz BM Hanni R Beng TS Huat TC Laboratory and field evaluation of the insect growth regulator pyriproxyfen (Sumilarv 0.5G) against dengue vectors J Am Mosq Control Assoc 2005 21 296 300 10.2987/8756-971X(2005)21[296:LAFEOT]2.0.CO;2 16252520

[B45] Yapabandara AM Curtis CF Laboratory and field comparisons of pyriproxyfen, polystyrene beads and other larvicidal methods against malaria vectors in Sri Lanka Acta Trop 2002 81 211 223 10.1016/S0001-706X(01)00208-X 11835898

[B46] Kamal HA Khater EIM The biological effects of the insect growth regulators; pyriproxyfen and diflubenzuron on the mosquito Aedes aegypti J Egypt Soc Parasitol 2010 40 565 574 21268527

[B47] Loh PY Yap HH Laboratory studies on the efficacy and sublethal effects of an insect growth regulator, pyriproxyfen (S-31183) against Aedes aegypti (Linnaeus) Trop Biomed 1989 6 7 12

[B48] Itoh T Kawada H Abe A Eshita Y Rongsriyam Y Igarashi A Utilization of bloodfed females of Aedes aegypti as a vehicle for the transfer of the insect growth regulator pyriproxyfen to larval habitats J Am Mosq Control Assoc 1994 10 344 347 7807075

[B49] Sihuincha M Zamora-Perea E Orellana-Rios W Stancil JD Lopez-Sifuentes V Vidal-Ore C Devine GJ Potential use of pyriproxyfen for control of Aedes aegypti (Diptera: Culicidae) in Iquitos, Peru J Med Entomol 2005 42 620 630 10.1603/0022-2585(2005)042[0620:PUOPFC]2.0.CO;2 16119551

[B50] Chavasse DC Lines JD Ichimori K Majala AR Minjas JN Marijani J Mosquito control in Dar es Salaam. II. Impact of expanded polystyrene beads and pyriproxyfen treatment of breeding sites on Culex quinquefasciatus densities Med Vet Entomol 1995 9 147 154 10.1111/j.1365-2915.1995.tb00171.x 7787222

[B51] WHO Pyriproxyfen in drinking-water: Use for vector control in drinking-water sources and containers 2008 Geneva: Background document for development of WHO guidelines for drinking-water quality

[B52] Mulla MS Darwazeh HA Kennedy B Dawson DM Evaluation of new insect growth regulators against mosquitoes with notes on nontarget organisms J Am Mosq Control Assoc 1986 2 314 320 2906977

[B53] Schaefer CH Miura T Dupras EF Mulligan FS Wilder WH Efficacy, nontarget effects, and chemical persistence of S-31183, a promising mosquito (Diptera: Culicidae) control agent J Econ Entomol 1988 81 1648 1655 3216066 10.1093/jee/81.6.1648

[B54] Schaefer CH Dupras EF Jr Mulligan FS III Studies on the environmental persistence of S-31183 (Pyriproxyfen): adsorption onto organic matter and potential for leaching through soil Ecotoxicol Environ Saf 1991 21 207 214 10.1016/0147-6513(91)90022-H 1676671

[B55] Sullivan J Environmental fate of pyriproxyfen[http://www.cdpr.ca.gov/docs/emon/pubs/fatememo/pyrprxfn.pdf]

[B56] Schaefer CH Miura T Chemical persistence and effects of S-31183, 2-(1-Methy-2(4- phenoxyphenoxy)ethoxy) pyridine on aquatic organisms in field tests J Econ Entomol 1990 83 1766 1776

[B57] WHO Guidelines for laboratory and field testing of mosquito larvicides 2005 Geneva: World Health Organization Communicable Disease Control, Prevention and Eradication. WHO Pesticide Evaluation Scheme. WHO/CDS/WHOPES/GCDPP/2005.2013

[B58] Kawada HD Dida GO Ohashi K Komagata O Kasai S Tomita T Sonye G Maekawa Y Mwatele C Njenga SM Mwandawiro C Minakawa N Takagi M Multimodial pyrethroid resistance in malaria vectors, Anopheles gambiae s.s., Anopheles arabiensis, and Anopheles funestus s.s. in Western Kenya PLoS One 2011 6 e22574 10.1371/journal.pone.0022574 21853038 PMC3154902

[B59] Araujo MS Gil LHS e-Silva AA Larval food quantity affects development time, survival and adult biological traits that influence the vectorial capacity of Anopheles darlingi under laboratory conditions Malar J 2012 11 261 10.1186/1475-2875-11-261 22856645 PMC3469369

[B60] Abbott WS A method of computing the effectiveness of an insecticide J Am Mosq Control Assoc 1987 3 302 303 3333059

[B61] Katz MH Multivariable analysis: A practical guide for clinicians 2006 2 Cambridge: Cambridge University Press

[B62] Marchand RP A new cage for observing mating behaviour of wild Anopheles gambiae in the laboratory J Am Mosq Control Assoc 1985 1 234 236 3880236

[B63] Becker N Rettich F Protocol for the introduction of new Bacillus thuringiensis israelensis products into the routine mosquito control program in Germany J Am Mosq Control Assoc 1994 10 527 533 7707059

[B64] Sumilarv, manufacturer’s product information[http://www.olyset.net/vectorcontrol/sumilarv/]

[B65] Kawada Y Shono Y Ito T Abe Y Laboratory evaluation of insect growth regulators against several species of anopheline mosquitoes Jap J Sanit Zool 1993 44 349 353

[B66] El-Shazly MM Refaie BM Larvicidal effect of the juvenile hormone mimic pyriproxyfen on Culex pipiens J Am Mosq Control Assoc 2002 18 321 328 12542190

[B67] Andrighetti MTM Cerone F Rigueti M Galvani KC Macoris MLG Effect of pyriproxyfen in Aedes aegypti populations with different levels of susceptibility to the organophosphate temephos Dengue Bulletin 2008 32 186 198

[B68] Hatakoshi M Kawada H Nishida S Kisida H Nakayama I Laboratory evaluation of 2-[1-methyl-2-(4-phenoxyphenoxy)-ethoxy] pyridine against larvae of mosquitoes and housefly Jpn J Sanit Zool 1987 38 271 274

[B69] Ali A Chowdhury MA Hossain MI Mahmud Ul A Habiba DB Aslam AF Laboratory evaluation of selected larvicides and insect growth regulators against field-collected Culex quinquefasciatus larvae from urban Dhaka, Bangladesh J Am Mosq Control Assoc 1999 15 43 47 10342267

[B70] Al-Sarar AS Al-Shahrani D Bayoumi AE Abobakr Y Hussein HI Laboratory and field evaluation of some chemical and biological larvicides against Culex spp. (Diptera: Culicidae) immature stages Int J Agr Biol 2011 13 115 119

[B71] Kawada H Dohara K Shinjo G Laboratory and field evaluation of an insect growth regulator, 4-phenoxyphenyl (RS)-2-(2-pyridyloxy) propyl ether, as a mosquito larvicide Jap J Sanit Zool 1988 39 339 346

[B72] Caputo B lenco A Cianci D Pombi M Petrarca V Baseggio A Devine GJ della Torre A The “auto-dissemination” approach: a novel concept to fight Aedes albopictus in urban areas PLoS One 2012 6 e1793 10.1371/journal.pntd.0001793 PMC3429402 22953015

[B73] Ali A Nayar JK Xue RD Comparative toxicity of selected larvicides and insect growth regulators to a Florida laboratory population of Aedes albopictus J Am Mosq Control Assoc 1995 11 72 76 7616194

[B74] Nayar JK Ali A Zaim M Effectiveness and residual activity comparison of granular formulations of insect growth regulators pyriproxyfen and s-methoprene against Florida mosquitoes in laboratory and outdoor conditions J Am Mosq Control Assoc 2002 18 196 201 12322941

[B75] Itoh K Control of DF/DHF vector, Aedes mosquito, with insecticides Trop Med 1993 35 259 267

[B76] Ye-Ebiyo Y Pollack RJ Kiszewski A Spielman A Enhancement of development of larval Anopheles arabiensis by proximity to flowering maize (Zea mays) in turbid water and when crowded Am J Trop Med Hyg 2003 68 748 752 12887038

[B77] Gimnig JE Ombok M Kamau L Hawley WA Characteristics of larval anopheline (Diptera: Culicidae) habitats in western Kenya J Med Entomol 2001 38 282 288 10.1603/0022-2585-38.2.282 11296836

[B78] Gimnig JE Ombok M Otieno S Kaufman MG Vulule JM Walker ED Density-dependent development of Anopheles gambiae (Diptera: Culicidae) larvae in artificial habitats J Med Entomol 2002 39 162 172 10.1603/0022-2585-39.1.162 11931252

[B79] Kaufman MG Wanja E Maknojia S Bayoh NM Vulule JM Walker ED Importance of algal biomass to growth and development of Anopheles gambiae larvae J Med Entomol 2006 43 669 676 10.1603/0022-2585(2006)43[669:IOABTG]2.0.CO;2 16892623

[B80] Mulligan FS III Schaefer CH Efficacy of a juvenile hormone mimic, pyriproxyfen (S-31183) for mosquito control in dairy waste water lagoons J Am Mosq Control Assoc 1990 6 89 92 2324729

[B81] Mohsen ZH Zayia HH Long-term sublethal effects of fenoxycarb against Culex mosquitoes (Diptera: Culicidae) Jpn J Sanit Zool 1995 46 151 154

[B82] Gaugler R Suman D Wang Y An autodissemination station for the transfer of an insect growth regulator to mosquito oviposition sites Med Vet Entomol 2012 26 37 45 10.1111/j.1365-2915.2011.00970.x 21689125

[B83] Vasuki V Influence of IGR treatment on oviposition of three species of vector mosquitoes at sublethal concentrations Southeast Asian J Trop Med Public Health 1999 30 200 203 10695811

[B84] Ohashi K Nakada K Miyaguchi J Shono Y Lucas JR Mito N Efficacy of pyriproxyfen-treated nets in sterilizing and shortening the longevity of Anopheles gambiae (Diptera: Culicidae) J Med Entomol 2012 49 1052 1058 10.1603/ME12006 23025186

[B85] Judson CL de Lumen HZ Some effects of juvenile hormone and analogues on the ovarian follicles of the mosquito Aedes aegypti (Diptera: Culicidae) J Med Entomol 1976 13 197 201 978693 10.1093/jmedent/13.2.197

[B86] Fournet F Sannier C Monteny N Effects of the insect growth regulators OMS 2017 and diflubenzuron on the reproductive potential of Aedes aegypti J Am Mosq Control Assoc 1993 9 426 430 8126477

[B87] Crowder DW Ellers-Kirk C Yafuso CM Dennehy TJ Degain BA Harpold VS Tabashnik BE Carriere Y Inheritance of resistance to pyriproxyfen in Bemisia tabaci (Hemiptera: Aleyrodidae) males and females (B Biotype) J Econ Entomol 2008 101 927 932 10.1603/0022-0493(2008)101[927:IORTPI]2.0.CO;2 18613596

[B88] Karatolos NK Williamson MS Denholm I Gorman K Richard H-C Bass C Over-expression of a cytochrome P450 is associated with resistance to pyriproxyfen in the greenhouse whitefly Trialeurodes vaporariorum PLoS One 2012 7 e31077 10.1371/journal.pone.0031077 22347432 PMC3275616

